# Honey bee (*Apis mellifera*) size determines colony heat transfer when brood covering or distributed

**DOI:** 10.1007/s00484-022-02308-z

**Published:** 2022-06-16

**Authors:** Derek Morville Mitchell

**Affiliations:** Institute of Thermofluids, School of Mechanical Engineering, University of Leeds, Leeds, UK

**Keywords:** CFD, Thermofluids, Acclimation, Climate change, Heat transfer

## Abstract

Heat transfer is key to the survival of honey bee colonies (*Apis mellifera* L.) in the wide range of hot (e.g. sub-Saharan) and cool climates (e.g. maritime-temperate) in which they have evolved and adapted. Here, a validated computational fluid dynamics, conjugate heat transfer model was used to determine the heat transfer of honey bee colonies in simulated standard wooden hives, complete with combs and brood, for a broad range of honey bee sizes, from slender lowland African *A.m. scutellata*, to broader (larger diameter) Northern European *A.m. mellifera*, across the whole range of brood covering honey bee densities, as well as when evenly distributed throughout the hive. It shows that under cooling stress, brood covering, broad subspecies need less than a third of the number of bees per unit of brood area for thermal insulation compared to slender subspecies. Also, when distributed evenly around the nest, broad subspecies lose less brood heat than when brood covering. These simulations demonstrate that honey bee girth has climate-based evolutionary advantages directly for the colony as well as via the survival of the individual. In addition, it shows that non-clustering behavioural patterns of passive honey bees can make significant, subspecies distinctive changes to nest heat loss and therefore honey production and climate change survival.

## Introduction

Computational fluid dynamics (CFD), through the simulation of honey bee nests, their environment and their occupants, can provide insights into multiple subspecies, across multiple climates, which would be difficult and costly, if not impossible, by other means. The goal of the study is to use CFD to simulate the impact of the differing body sizes of the honey bee subspecies on heat transfer from brood areas, in commercially available hives. This research uses the power of CFD to explore both the conventional (e.g. honey bees do not heat the hive (Farrar 1952)⁠) and alternative hypotheses (e.g. honey bee size is significant factor for hive convection⁠) of temperature and heat transfer-related honey bee behaviour by considering a much wider ranging combination of states, ambient temperatures, colony sizes and subspecies than are logistically possible with conventional animal experiments.

By simulating a very small subset of the possible honey bee behaviours (generation of heat in the brood region and passive obstruction of air flow) in a wide range of condition combinations, this research aims to show how the honey bee and its environment interact at a basic level and thus form the basis to understand the intent and results of their more complex behaviours.

This study simulates the different subspecies in identical volume nests/hives but using the bee number densities for the subspecies derived from the literature (Schneider and Blyther 1988; McNally and Schneider 1996; Saucy 2014; Mulisa et al. 2018)⁠. This commercially important pollinator has evolved several (circa 24) subspecies suited to diverse environments from tropical forests and semi-desert to temperate lands that have − 40C winters. These subspecies vary in body diameter and body hair length (Ruttner 1988)⁠ demonstrating an increase in both in colder climates. They have also evolved behaviours for selecting and manipulating (Seeley 1985)⁠ their nest thermofluid environment including nest selection for thermal performance, close temperature regulation in brood area via endothermy and advection, evaporation of large volumes of liquid (nectar to honey 200 + kg per year) and the resulting water vapour transport (Mitchell 2019) and clustering to reduce heat losses.

The ability to withstand changes in ambient temperature without resorting to torpor by clustering in colder climates, in winter inside the nest and in spring outside while swarming, has been related in extensive studies into the thermography (Kovac et al. 2009; Stabentheiner et al. 2021)⁠⁠ and metabolic rates (Southwick 1982)⁠. There have also been studies into the theoretical heat transfer in a clustered state outside of the nest (Myerscough 1993; Watmough and Camazine 1995; Basak et al. 1996; Ocko and Mahadevan 2014)⁠, a convective computational fluid dynamic (CFD) analysis of honey bees clustered in a hive (Sudarsan et al. 2012; Thompson 2013)⁠, conductive heat transfer on individual combs (Humphrey and Dykes 2008)⁠ and experimental studies on the lumped conductance of the nest enclosure (Mitchell 2016)⁠. However, there has not been, to date, a study which takes account of the following: first, both the convective and conductive heat transfer of complete honey bee nests/hives; second, the contribution of those honey bees not clustered and ectothermic by passively resisting air movement; and third, differences arising from honey bee size or differences arising from honey bee density.

In the most comprehensive CFD study of hive air flow to date (Sudarsan et al. 2012), convective airflow through the honey bees was treated as flow through an inert porous material of small cylinders in a perfectly insulated hive. However, that CFD study considered a single fixed honey bee size (11 mm length, 3 mm dia) from an unpublished estimate and then derived the narrow porosity range (0.4–0.5) from published known cluster sizes and populations (Heinrich 1981)⁠. This makes the calculations extremely sensitive to the honey bee diameter used (Eqs. 1, 2 and Table 1). In the current study, this sensitivity is overcome by both the porosity and honey bee size being treated as independent variables over as wide a range as practicable. This allowed the study of the wide variety of *Apis mellifera* subspecies adapted to various climates in Africa and Europe which exhibit different physical characteristics in both their phenotype, i.e. body size, and extended phenotype, i.e. their nest.

**Table 1:** Symbols

Symbol	Units	Description
$\nabla P$	N m^−3^	Pressure differential per unit length
$\overrightarrow{U}$	ms^−1^	Velocity
$\beta$	kgm^−4^	2nd order velocity coefficient (impact)
$\alpha$	Nm^−4^ s	1st order velocity coefficient (viscous drag)
$\mu$	Nm^−2^ s	Dynamic viscosity
$\varphi$	-	Porosity
${d}_{P}$	m	Particle diameter
$\rho$	kgm^−3^	Density
$\overline{d}$	m	Generic effective diameter
${d}_{F}$	m	Effective particle diameter after Sudarsan et al. (2012)⁠
${V}_{P}$	m^3^	Volume of particle
$\psi$	-	Shape factor
${A}_{P}$	m^2^	Surface area of particle
${d}_{SD}$	m	Sauter mean value
${d}_{EQ}$	m	Effective particle diameter after Li and Ma (2011)⁠
${d}_{oriface}$	m	Effective diameter of mesh opening
$L$	m	Depth of mesh in simulation
${\rho }_{B}$	m^−3^	Number of honey bees per unit volume
${V}_{Bee}$	m^3^	Average volume of individual honey bee
${l}_{Bee}$	m	Length of honey bee

The volume of the natural nest, and the number of honey bees in them, vary on a subspecies basis, but the number of honey bees per unit volume are within 10% of each other (Table 2) (Schneider and Blyther 1988; Saucy 2014; Mulisa et al. 2018)⁠ at around 1.3 × 10^6^ honey bees per m^3^. The studies in honey bee taxonomy to date have not focused on body length and diameter, with or without body hair, so the exact dimensions are uncertain and are further complicated by anthropogenic size modification (Saucy 2014). However, the range can be implied from the comb cell size in which the honey bees naturally pupate, and enter to clean etc. The cell sizes are shown in Table 2. The upper bound has been validated in this study by a limited photometric survey by the author of hybridised European bees located in the UK.

**Table 2:** Subspecies cell sizes, colony populations and volumes (Schneider and Blyther 1988; Saucy 2014; Mulisa et al. 2018)⁠

	Parameter	*A.m. scutellata*	European
1	Nest volume m^3^ 10^−3^	17	45
2	Population 10^3^	6.4	18.8
3	Cell diameter m 10^−3^	2.5–4.3	4.4–5.5
4	Cell length m 10^−3^	9.5–11.4	11–12
5	Inter-comb gap (bee space) m 10^−3^	9–11	9–11
6	Total inter-comb volume m^3^ 10^−3^	5.1	13.5
7	Individual honey bee volume m^3^ 10^−9^	54–138	167–261
8	Distributed bee number density in inter-comb volume m^−3^ 10^6^	1.25	1.39
9	Distributed bee volume density in inter-comb volume m^3^ m^−3^	0.07–0.18	0.23–0.36
10	Distributed bee porosity of inter-comb volume	0.82–0.93	0.64–0.77
11	Brood area m^2^	0.24	0.59
12	Bees per unit area of brood m^−2^ 10^3^	17.3–40.2	24.1–42.4

For a given nest volume, the volume surrounding the comb in which the adult honey bees reside (inter-comb volume) is, from comb geometry, nearer 0.3 of the total, and this is the volume used to calculate the porosity.

### Thermofluid modelling

To understand the significance of these differences in heat transfer, we need to look into the fluid dynamic theory related to porous materials.

The pressure differential caused by porous media such as distributed insects is the Darcy-Forchheimer model of pressure difference per unit length across a porous material (Nield and Bejan 2006)⁠:

1 $$\nabla\mathrm P=-\mathrm\alpha\overrightarrow{\mathrm U}-\mathrm\beta\left|\overrightarrow{\mathrm U}\right|\overrightarrow{\mathrm U}$$

The first term in Eq. 1 relates to the viscous drag and is the dominant term below Reynolds numbers of 10. The second term relates to the obstruction effects of the particles.

Ergun’s equation formulates α and β as per Eqs. 2 and 3 (Li and Ma 2011):⁠

2 $$\mathrm\alpha=\mathrm\mu150\frac{\left(1-\mathrm\varphi\right)^2}{\mathrm d_{\mathrm P}^2\mathrm\varphi^3}$$

3 $$\mathrm\beta=\frac{\mathrm\rho}2\frac{3.5\left(1-\mathrm\varphi\right)}{{\mathrm d}_{\mathrm P}\mathrm\varphi^3}$$

But this is not valid for particles that deviate strongly from a spherical shape or have large size distributions. So d_P_ is replaced by an effective particle diameter d usually calculated from the relationship between the volume and area (Eqs. 4 and 5).

4 $$\mathrm\alpha=\mathrm\mu150\frac{\left(1-\mathrm\varphi\right)^2}{{\mathrm {\overline d}^2}\mathrm\varphi^3}$$

5 $$\mathrm\beta=\frac{\mathrm\rho}2\frac{3.5\left(1-\mathrm\varphi\right)}{\overline{\mathrm d}\mathrm\varphi^3}$$

Previous workers (Basak et al. 1996; Sudarsan et al. 2012)⁠ have used an effective particle diameter d_F_ (Eq. 6) then apply a shape factor Ψ (Eq. 7) which gives a result equal to the Sauter mean value d_SD_ (Eq. 8).

6 $${\mathrm d}_{\mathrm F}=\left(6{\mathrm V}_{\mathrm P}\right)^\frac13\mathrm\pi^{-\frac13}$$

7 $$\mathrm\psi=\frac{\mathrm{\pi}d_{\mathrm F}^2}{{\mathrm A}_{\mathrm P}}=\frac{\mathrm\pi^\frac13\left(6{\text{V}}_{\mathrm P}\right)^\frac23}{{\mathrm A}_{\mathrm P}}$$

8 $$\overline{\mathrm d}={\mathrm{\psi}d}_{\mathrm F}$$

9 $$\overline{\mathrm d}={\mathrm d}_{\mathrm{SD}}=\frac{6{\text{V}}_{\mathrm P}}{{\mathrm A}_{\mathrm P}}$$

One experimental engineering study (Li and Ma 2011) used cylinders quite close to the honey bee in terms of scale and aspect ratio, i.e. cylinders 6mm long and 3mm diameter. They determined that a more accurate approximation was to use the product of the shape factor and the Sauter mean diameter (Eq. 10).

10 $$\overline{\mathrm d}={\mathrm d}_{\mathrm{EQ}}=\frac{\mathrm\pi^\frac13\left(6{\text{V}}_{\mathrm P}\right)^\frac53}{\mathrm A_{\mathrm P}^2}$$

To use Eq. 10, we need to compute the porosity of the different honey bees in their respective nests when distributed evenly around the nest. From the geometry of the comb and inter-comb spaces, we can determine the non-comb, honey bee occupied volume of the nest and hence the number density of the honey bees (ρ_B_). This, with the volume of the individual honey bee (V_bee_), can be used in Eq. 11 to give the porosity.

11 $$\mathrm\varphi=1-{\mathrm\rho}_{\mathrm B}{\mathrm V}_{\mathrm{Bee}}$$

To understand the impact of species differences, the variation of the α and β coefficients need to be known and their consequent changes to the overall heat transfer determined.

## Methods

In order to determine the significance of differences in air resistances to the heat lost from a hive, a CFD simulation of full conjugate heat transfer was conducted of *Apis mellifera* colonies in a standard hive, complete with combs and brood.

**Fig. 1:**
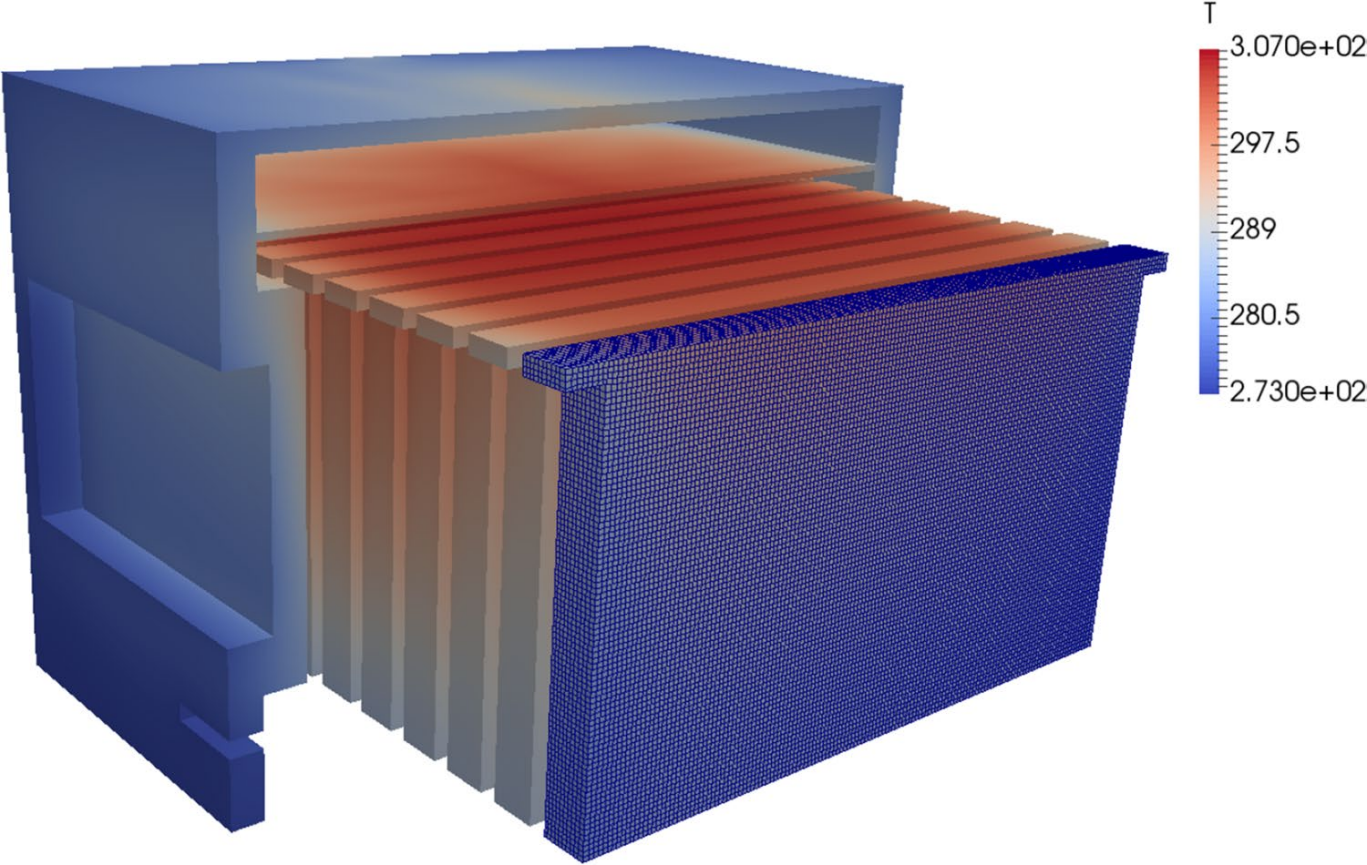
Cutaway of CFD model showing combs and typical temperature distribution and meshing

**Fig. 2:**
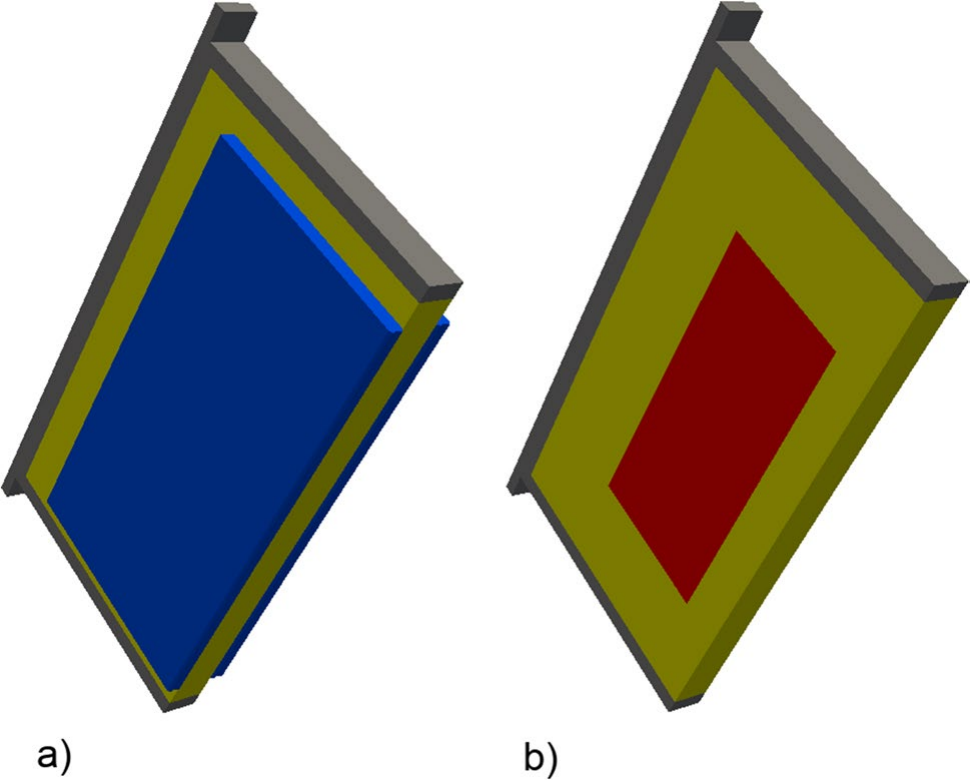
CFD brood comb frames, with covering honey bees (**a**) and without (**b**) colour code: frame as grey, comb as yellow, brood covering honey bees as blue and brood cells as red

A computer-aided design model (CAD) model of a standard British National Hive (Cushman 2011)⁠ of approximately 35-L capacity was constructed using FreeCAD (Riegel and Mayer 2019)⁠. The dimensions were taken from an example Western Red Cedar hive and combs supplied by Thornes Ltd. The model simulated 12 standard combs, empty of stores or brood (Fig. 1) except for the 6 central combs, each of which had brood areas of approximately 214 by 100 mm (Fig. 2a). This model contained air surrounding the hive (1 m by 1 m by 2 m), mesh floor, entrance, crown board, roof and ventilated roof cavity as well as the internal air volume occupied by honeybees while distributed about the nest. For the clustered data, the brood covering volumes 304 × 140 × 10 mm were simulated (Fig. 2b). This model was then loaded into the CFD tool OpenFOAM v4.1. (Jasak et al. 2007)⁠. The comb thickness and inter-comb space were fixed at 25 mm and 10 mm, respectively.

### Assumptions

The following assumptions were made in the CFD modelling:

- The honey bee colony is in either one of two states: first, honey bees covering the thermoregulated honey bee brood (*brood covering*) and, second, evenly distributed around the hive (*distributed*). Conventionally, the *distributed* state can be thought to represent the summer day time or warm climate configuration and the *brood covering* state the colder climate or winter configuration.
- The honey bees are approximated to cylinders 11 mm long and diameters as specified.
- The volume of brood on each of the six simulated brood containing combs is fixed, rectilinear and isothermally maintained at 307 K and is the only heat source within the nest, i.e. the contribution from endothermic bees is considered to be located either on the brood surface or within the brood (brood volume).
- Radiation is ignored for the purposes of this simulation.
- The flow is transitional from laminar to turbulent and thus amenable to a kwSST turbulence model.
- Condensation and evaporation are ignored.
- Thermal conductance of honey bee bodies is ignored.
- A single fluid, air, is considered.
- For the *distributed* state, all of the colony bees are simulated as evenly distributed into the space within 10 mm of all the internal structures, i.e. a fixed volume of 10.5 L.
- In the *brood covering* state, all of the honey bees in the colony are simulated as evenly distributed in 7 fixed brood covering volumes 304 × 160 × 10 mm on each face of the 6 brood areas, i.e. a fixed volume of 3.4 L. Thus, varying porosity and number density values represent colonies with differing numbers of honey bees.
- The lower limit of porosity, and the highest number density (e.g. when honey bees are clustered at extreme low temperatures), is the geometric limit of close packed cylinders, i.e. 0.095.

The model was meshed using the standard OpenFOAM mesh utility *snappyHexMesh*. Care was taken to ensure that the mesh was sufficiently fine in the boundary layers to enable valid lower Reynolds number turbulence modelling relevant to a kω-SST turbulence model (Menter et al. 2003; Moukalled et al. 2016)⁠ using the *y*^+^ metric. The meshing gave values of *y*^+^  ~ 10^−2^ (CFD-Online 2014a)⁠ inside and around the hive and 4 on the test cell walls. The meshed model was then used in the standard compressible flow, steady-state conjugate multi-region heat transfer solver, *CHTmultiRegionSimpleFoam*, with *kω-SST* turbulence using the boundary condition *turbulentTemperatureCoupledBaffleMixed*, for coupling between the solid and fluid regions of the hive. The boundary conditions, at the walls of the volume under test, were set to fixed zero velocity gradient, with the inlet turbulent energy, turbulent dissipation rate and specific turbulent dissipation rate set to fixed values according to the literature (CFD-Online 2014b). The fixed parameters in Table 3 were used in the simulation.

**Table 3:** CFD parameters

	Parameter	Value	Units
1	Empty comb conductance	0.023	WK^−1^ m^−1^
2	Ambient air velocity (inlet)	0.05	ms^−1^
3	Inlet turbulent energy k	9.79 10^−8^	m^2^s^−2^
4	Inlet turbulent dissipation rate ε	9.94 10^−11^	M^2^s^−3^
5	Inlet-specific turbulent dissipation rate ω	3.38 10^−3^	s^−1^
6	Comb frame conductance	0.12	WK^−1^ m^−1^
6	Hive conductance	0.12	WK^−1^ m^−1^
8	Wire mesh pitch	4	mm
9	Wire mesh wire diameter	1	mm
10	Brood covering volume	3.4	Litres
11	Model mesh size	3.2	Cells × 10^6^

The empty comb conductance was selected to be close to that used by other workers (Humphrey and Dykes 2008) and also suitable for later validation.

The ambient air velocity was chosen after sensitivity testing for a combination of rapid CFD solving and minimal impact on conductance, i.e. less than 3%.

### Wire mesh and honey bee porosity modelling

In order to simulate the wire mesh, the industry standard formulae (Idelchik 2006)⁠ were used to derive the *α* and *β* coefficients.⁠

12 $$\alpha =\mu \frac{11\varphi }{{d}_{orifice}L}$$

13 $$\mathrm\beta=\frac{\mathrm\rho}2\frac1{\mathrm L}\left(1.3\left(1-\mathrm\varphi\right)+\left(\frac1{\mathrm\varphi}-1\right)^2\right)$$

Both the honey bee volume and the wire mesh *α* and *β* coefficients were input into the model as parameters for porosity zones in the air region. This was accomplished using the explicit porosity *fvOption* facility within OpenFoam to modify the governing equations for momentum in the specified zones in order to implement Eq. 1. The thermoregulated brood was emulated as a zone of fixed temperature within the comb region using the explicit heat source *fvOption* facility to modify the governing equations for enthalpy in the specified zones.

The differing zones of thermal conductance within the comb regions were implemented by an enthalpy modification field to scale the conductance to an appropriate level from a generic comb value.

### Execution

A separate CFD run was conducted for each combination of the following:

- Honey bee effective diameter
- Honey bee porosity
- *Brood covering* or *distributed* states
- Ambient temperature

The iteration steps were continued until the temperatures within the model reached equilibrium, typically after 3500 iterations.

### Post processing

The heat flux from the frames into the surrounding air was computed from each of the runs using the *wallHeatFlux* (Venkatesh 2016) post processing function. In addition *Paraview* (Ayachit 2015) was used to derive visualisations of temperature and air flow. The results along with the key parameters were loaded into an open source SQL database (MariaDB (Widenius 2020)) and then plotted using *MATlab* (MATLAB 2018).

### Validation

The CFD was validated by first validating the CFD model convection/conduction resistance against a physical experiment; second, validating the porosity pressure differential against published results; and, third, a mesh sensitivity analysis.

The physical experiment used the hive that provided the dimensions for the model. The thermoregulated brood in each comb was emulated by 6 pairs of 12-mm-thick electrically heated, temperature-monitored aluminium plates. The empty comb was emulated by 25-mm-thick polyisocyanurate (PIR) insulation.

## Results

### Parameter analysis

Given the complexity of the problem, it is useful to understand how key parameters interact before interpreting the CFD results. The resistance coefficients, *α* and *β*, to convective airflow are derived from the resistance of spheres, by using a conversion from the cylinder dimensions to the diameter of a sphere of the same effective resistance (see effective diameter in Eq. 10).

**Fig. 3:**
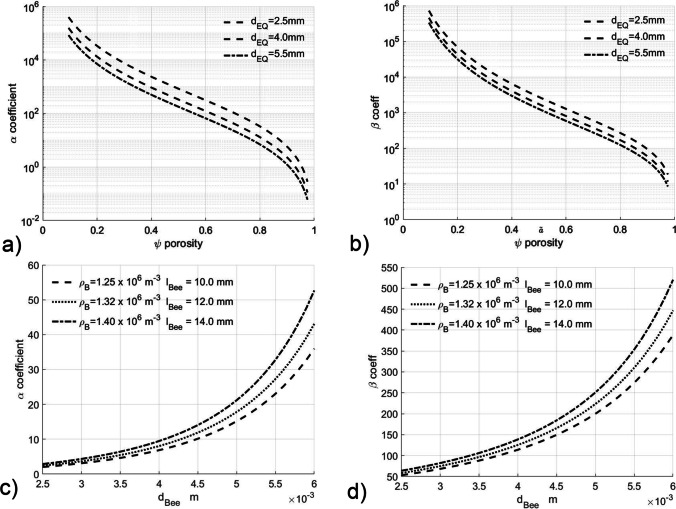
α and β air flow resistance coefficients versus **a** and **b** porosity Ψ for varying effective diameters (d_EQ_) and **c** and **d** actual bee diameters d_Bee_ at constant bee number densities ρ_B_

The variations of the *α* and *β* coefficients versus honey bee porosity for constant values of honey bee effective diameter as used in the CFD runs are plotted in Fig. 3a and b and versus actual honey bee diameter at values of constant number density and actual honey bee length in Fig. 3c and d.

### CFD results

**Fig. 4:**
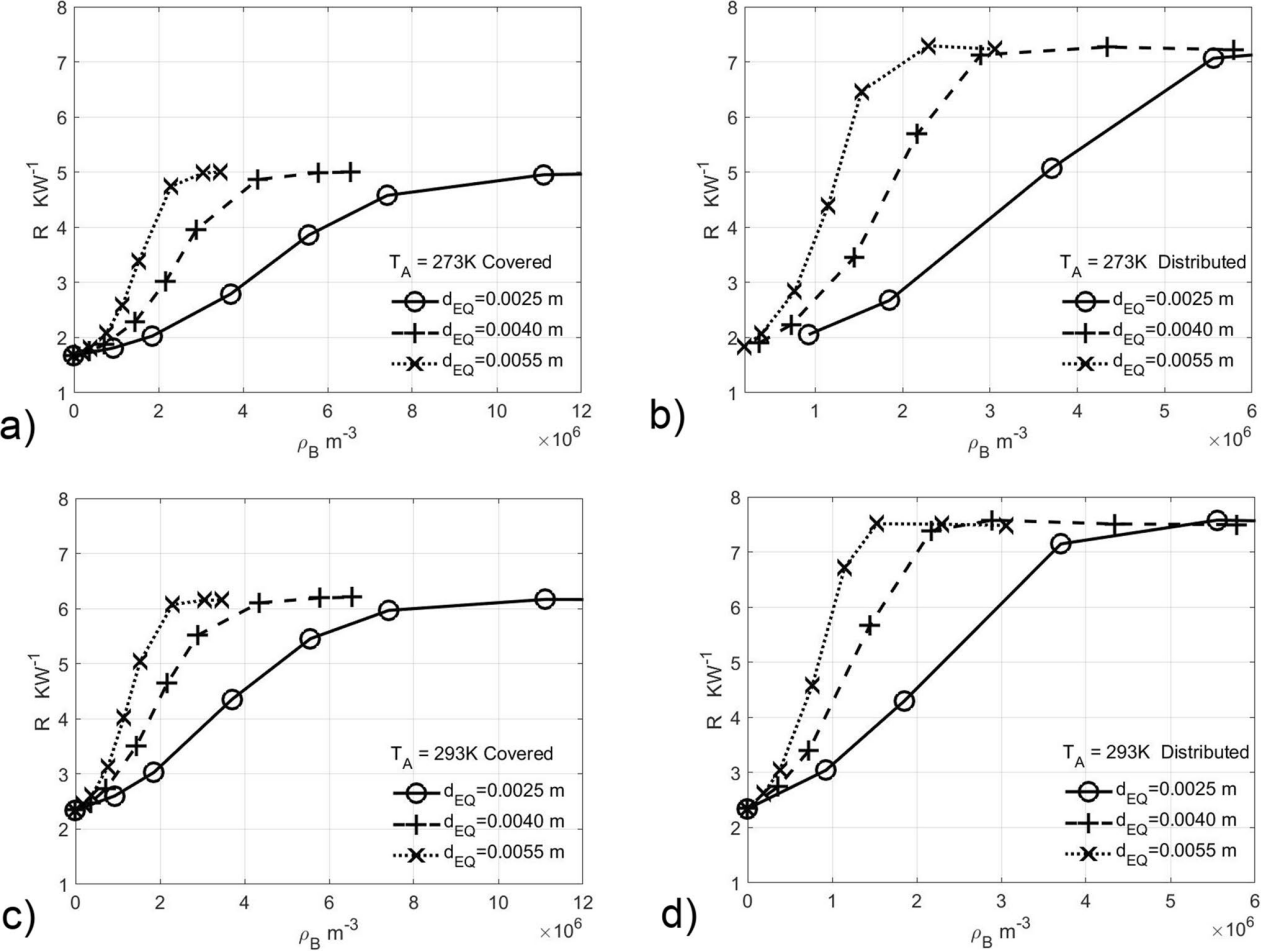
Brood covering and distributed hive thermal resistance vs colony number density for effective diameters at constant ambient temperatures **a** 273 K brood covering, **b** 273 K distributed, **c** 293 K Brood covering and **d** 293 K distributed. The rightmost termination of the lines for distributed indicates the geometric packing limit with the exception of 2.5 mm diameter between

**Fig. 5:**
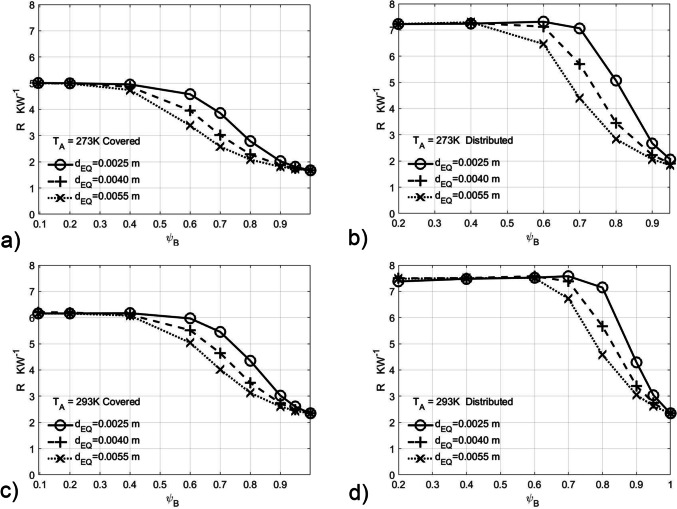
Brood covering and distributed hive thermal resistance vs porosity for constant effective diameters at constant ambient temperatures **a** 273 K brood covering, **b** 273 K distributed, **c** 293 K brood covering and **d** 293 K distributed

For the *brood covering* state, the CFD experiment used the porosity range 0.095 to 1.0 and the *distributed* state a range 0.2 to 1.0, both at temperatures of 273 and 293 K. The plot of hive thermal resistance vs honey bee number density at constant ambient temperatures and effective diameter size for both *brood covering* & *distributed* is shown in Fig. 4. The plot of hive thermal resistance vs porosity at constant ambient temperatures and effective diameter size for both *brood covering* & *distributed* is shown in Fig. 5.

## Discussion

From Eqs. 1, 2, and 3, we can see that the key factors determining air resistance, and hence heat transfer are the porosity and the effective diameter of the honey bees. The length of the subspecies varies by about 10% and cause only minor changes in effective diameter. However the difference in diameter is in excess of a factor of 2 and in volume by a factor of 5. In contrast the bee number density only changes by 10%. This leads to a difference in porosity when the honey bee are evenly distributed around the nest, with ~ 95% for low-land *A.m. scutellata* (effective diameter ~ 2.5 mm) compared to ~ 70%, for European honey bees (effective diameter ~ 5 mm, from Table 2).

Previous studies (Sudarsan et al. 2012), which concentrated on porosities between 0.4 and 0.5, can now be shown to reside in the region where the thermal resistance is already at its maximum (Fig. 5).

From Fig. 3a and b, it can be clearly seen that such an apparently small difference in porosities will lead to a marked difference in air resistance coefficients α and β (factor of ~ 20) and hence thermal resistance. Similarly, at constant number densities, changes in diameter of 2.5 to 5.0 mm lead to coefficient changes of a factor of 7 (Fig. 3c and d). This is borne out by the differences in thermal resistance (Fig. 4) between subspecies at the same number density in both *brood covering* and *distributed* CFD simulations across all temperatures, with the difference of approximately a factor of 2 in thermal resistance between the most extreme African and European subspecies (Fig. 4b and c) at the observed distributed honey bee densities (1.25 and 1.39 × 10^6^ m^−3^ from Table 2).

This higher thermal resistance of the colony for broad honey bees might be seen as part of the adaptation of European honey bees to their colder climate by apparently conforming to Bergmann’s and Allen’s rules (Freckleton et al. 2003; Nudds and Oswald 2007). These state that body size and appendage width to length ratio of homeothermic animals increase inversely with climate temperature, because of the change in body surface area to volume ratio. While often reliable with mammals, it should be noted that these rules are not suitable for Hymenoptera (Shelomi 2012) or even social bees (Gérard et al. 2018). Given *Apis mellifera* are not homeotherms, can be either ectothermic or strongly endothermic and spend 80% + of their lifetime inside the nest, including the times of greatest seasonal or daily thermal stress, the individual’s body surface area to volume ratio is of diminished relevance and weakens Bergmann’s validity in honey bees. The heat loss causality for Allen’s rule is also weak for honey bees given that the heat source, i.e. muscles, is in the thorax. Reduced limb length is observed in more northerly bees (Ruttner 1988), and the resulting shortened gait may be interlinked with the higher number densities observed in northern honey bees. This is feasible if the honey bees were density regulated by the number of honey bee steps between honey bee to honey bee encounters, honey bee density sensitivity and step counting being known in other behaviours (Seeley 1977; Smith et al. 2017). The resulting higher number densities can then combine with girth to increase airflow resistance (Fig. 3c and  d).

The case for air flow resistance driving girth changes is further strengthened owing to the fact that the classic surface area to body ratio is a linear phenomenon, whereas resistance to nest airflow rises close to the cube of body diameter (Fig. 3c and  d).

It has been observed that the length of body hair on honey bees has a correlation with latitude (Ruttner 1988). The longer hair has been credited with giving greater heat retention when tightly clustered (Southwick 1983); however, it can be seen that the upper limit of thermal resistance is reached well before cylinder packing limit (Fig. 4). Therefore, the increase in hair length is not relevant to being tightly clustered but is useful in reducing the individuals and colonies heat loss, not only by impeding air flow (Bejan 1990) to the surface of the individual, e.g. outside the hive, but also by reducing the colonies porosity via increasing the honey bees individual volume. Thus, the very low porosity of winter clustering does not improve thermal resistance of the core endothermic honey bees but instead improves thermal contact between the individuals and the core. However, if thermal contact with other honey bees was dominant for evolution, then maximisation of surface area would occur, and a converse Bergmann relation would be observed in a similar manner to ectotherms needing surface area to gain heat from the sun.

The upper thermal resistance limit indicates the honey bee density at which the air stops moving under convection, and heat transfer is by conduction through the air either in the brood cover volume or the space occupied by the distributed honey bees. Thus, at the observed distributed number densities, we can see that broad subspecies are close to the thermal resistance limit, i.e. conduction only, while the slender subspecies are distant from the thermal resistance limit, and thus the interior is strongly convective. This is indicative of a strong adaptation to climate for active colonies, with the slender subspecies able to dissipate heat in a warm climate, and the broad species retain it in a cool climate.

A surprising result is the thermal resistance of a *distributed* colony at the observed distributed number density that is greater than thermal resistance limit for *brood covering* for the broad subspecies (Fig. 4a  and c) and the reverse for slender subspecies. While for broad subspecies in cool climates, the *distributed* state reduces heat loss, for the slender subspecies, in the *distributed* state, at lower ambient temperatures, individual honey bees near the periphery would fail to stay above fatal temperatures.

This suggests that the driver for broad subspecies to brood cover is not directly for colony survival by reducing colony heat loss but is driven by other behaviours and needs.⁠

In the active season, this closeness in broad subspecies to the upper thermal limit, when natural convection is close to being suppressed, implies that there are circumstances where honey bees will have to move to allow natural convection or forced convection to take place. Whereas studies of patterns of behaviour have previously been focused on action, e.g. endothermy in the brood area or storing pollen and nectar, it can now be seen that the location of inactive ectothermic honey bees can have a significant impact on the thermofluid processes in the nest. Evidence of this is apparent when honey bees come out of the nest en-mass and wait around the entrance, called “bearding” (Hamdan 2010)⁠. To date this has been labelled as “over heating” or “overcrowding” but may in fact be behavioural movements to allow air flow-based activity such as honey desiccation.

The role of the male bees, a larger diameter minority (approximately 20% and cell size > 6 mm) occupant of the nest (Seeley 1985)⁠, becomes of interest, particularly, since they have been observed to frequently congregate on outer frames of the nest, where their larger bodies, if at the same number density, would impart a stronger resistance to air flow down the cooler surfaces of the nest and hence present an effective thermal resistance. This is of relevance since male honey bees are present in the nest for large periods of the year when lower temperatures, e.g. at night, can be found. This is illustrated by *A.m. mellifera*, a large diameter subspecies, whose drones can be present from April to August and its external night time environment can be close to 0 C.

It is likely that there are other such behaviours that passively manipulate the airflow, not yet noted or studied.

For brood covering honey bees at ambient 293 K (Fig. 5c), if we consider the bee densities, where the limit of thermal resistance is reached, we can see it is markedly different for 2.5 mm and 5.5 mm diameter bees, i.e. ~ 11.5 and 3 × 10^6^ m^−3^. Thus, if thermal stress resistance is a limiting factor for brood production, then the broad subspecies can cover over 3 times the brood area compared to slender species for the same thermal stress. That both broad and slender have similar numbers of bees per unit area of brood (Table 2) may indicate the differences of thermal stress caused by the climates of the subspecies involved.

This study considers *brood covering* and *distributed* honey bee densities as isolated states, whereas in reality, they are a continuum bounded by the total colony population. Thus, there is further work to be done, e.g. using colony size and a *brood covering* to *distributed* blending function instead of honey bee number density.

## Conclusion

This study has shown the following:

- Body hair has an impact on colony heat transfer but not when tightly clustered.
- Body diameter halves brood heat loss for broad subspecies.
- Tight clustering is primarily for individual survival by thermal contact.
- “Doing nothing” ectothermic honey bees are reducing heat transfer especially when large diameter, i.e. drones.
- Honey bees gathering outside the hive, for broad subspecies, is a necessary method of increasing heat transfer and internal air transport.
- The normally assumed causation of the apparent conformity to Bergmann’s and Allen’s rules, where the ratio of individual surface area to individual volume determines size, is unreliable. Here it appears that it is the relation of colony number density to total colony body volume (i.e. individual girth) that is significant in determining the variation of body size with latitude.

These insights should influence how researchers and bee keepers interpret the acclimation and behaviour of the different subspecies of honey bees and consider patterns of distribution of relatively inactive honey bees as worthy of notice. It is clear that CFD can provide numerous new biological insights into multiple subspecies of honey bees across multiple climates, which would be difficult and costly, if not impossible, by other means, and provides a means of testing the physical validity of hypotheses of evolutionary pressure.

## References

[CR1] Ayachit U (2015) The ParaView guide: a parallel visualization application. Kitware, Incorporated

[CR2] Basak T, Rao KK, Bejan A (1996). A model for heat transfer in a honey bee swarm. Chem Eng Sci.

[CR3] Bejan A (1990). Theory of heat transfer from a surface covered with hair. J Heat Transfer.

[CR4] CFD-Online (2014a) Dimensionless wall distance (y plus) [Internet]. CFD-Online. [cited 2022 Feb 2]. Available from: https://www.cfd-online.com/Wiki/Dimensionless_wall_distance_(y_plus)

[CR5] CFD-Online (2014b) Turbulence free-stream boundary conditions [Internet]. CFD-Online [cited 2022 Feb 2]. Available from: https://www.cfd-online.com/Wiki/Turbulence_free-stream_boundary_conditions

[CR6] Cushman D (2011) Drawings of hives and hive parts. Dave Cushman’s Beekeeping and Bee Breeding Website

[CR7] Farrar CL (1952). Ecological studies on overwintered honey bee colonies. J Econ Entomol.

[CR8] Freckleton RP, Harvey PH, Pagel M (2003). Bergmann’s rule and body size in mammals. Am Nat.

[CR9] Gérard M, Vanderplanck M, Franzen M, Kuhlmann M, Potts SG, Rasmont P (2018). Patterns of size variation in bees at a continental scale: does Bergmann’s rule apply?. Oikos.

[CR10] Hamdan K (2010). The phenomenon of bees bearding. Bee World.

[CR11] Heinrich B (1981) The mechanisms and energetics of honeybee swarm temperature regulation. J Experiment Biol 91

[CR12] Humphrey JAC, Dykes ES (2008). Thermal energy conduction in a honey bee comb due to cell-heating bees. J Theor Biol.

[CR13] Idelchik IE (2006) Handbook of hydraulic resistance (3rd edition). Washington.

[CR14] Jasak H, Jemcov A, Tukovic Z (2007) OpenFOAM : A C ++ Library for complex physics simulations. Int Work Coupled Methods Numer Dyn. m:1–20

[CR15] Kovac H, Stabentheiner A, Brodschneider R (2009). Contribution of honeybee drones of different age to colonial thermoregulation. Apidologie.

[CR16] Li L, Ma W (2011). Experimental study on the effective particle diameter of a packed bed with non-spherical particles. Transp Porous Media.

[CR17] MATLAB. 9.4.0.813654 (2018) Natick, Massachusetts: The MathWorks Inc.

[CR18] McNally LC, Schneider SS (1996). Spatial distribution and nesting biology of colonies of the African honey bee Apis mellifera scutellata (Hymenoptera: Apidae) in Botswana. Africa Environ Entomol.

[CR19] Menter FR, Ferreira JC, Esch T (2003 ) The SST turbulence model with improved wall treatment for heat transfer predictions in gas turbines. Int Gas Turbine Congr [Internet] 1992:1–7. Available from: http://nippon.zaidan.info/seikabutsu/2003/00916/pdf/igtc2003tokyo_ts059.pdf

[CR20] Mitchell D (2016). Ratios of colony mass to thermal conductance of tree and man-made nest enclosures of Apis mellifera: implications for survival, clustering, humidity regulation and Varroa destructor. Int J Biometeorol.

[CR21] Mitchell D (2019). Nectar humidity, honey bees (Apis mellifera) and varroa in summer: a theoretical thermofluid analysis of the fate of water vapour from honey ripening and its implications on the control of Varroa destructor. J R Soc Interface.

[CR22] Moukalled F, Managai L, Dawish M (2016) The finite volume method in computational fluid dynamics. Heidelburg: Springer

[CR23] Mulisa F, Alemayehu A, Diribi M, Fekadu B, Alayu T (2018). Determination of bee spacing and comb cell dimensions for Apis mellifera Scutellata honeybee race in western Ethiopia. Int J Livest Prod.

[CR24] Myerscough MR (1993). A simple model for temperature regulation in honeybee swarms. J Theoretical Biol.

[CR25] Nield DA, Bejan A (2006) Convection in porous media. 3rd ed. Springer

[CR26] Nudds RL, Oswald SA (2007). An interspecific test of Allen’s rule: evolutionary implications for endothermic species. Evolution (n y).

[CR27] Ocko SA, Mahadevan L (2014). Collective thermoregulation in bee clusters. J R Soc Interface..

[CR28] Riegel J, Mayer W (2019) FreeCAD (Version 0.18)

[CR29] Ruttner F (1988) Biogeography and taxonomy of honeybees. Biogeography and Taxonomy of Honeybees

[CR30] Saucy F (2014). On the natural cell size of European honey bees: a “fatal error” or distortion of historical data?. J Apic Res.

[CR31] Schneider S, Blyther R (1988). The habitat and nesting biology of the African honey bee Apis mellifera scutellata in the Okavango River Delta, Botswana. Africa Insectes Soc.

[CR32] Seeley T (1977). Measurement of nest cavity volume by the honey bee (Apis mellifera). Behav Ecol Sociobiol.

[CR33] Seeley TD (1985). Honeybee ecology: a study of adaptation in social life.

[CR34] Shelomi M (2012). Where are we now? Bergmann’s rule Sensu Lato in insects. Am Nat.

[CR35] Smith ML, Koenig PA, Peters JM (2017). The cues of colony size: How honey bees sense that their colony is large enough to begin to invest in reproduction. J Exp Biol.

[CR36] Southwick EE (1982). Metabolic energy of intact honey bee colonies. Comp Biochem Physiol Part A Physiol.

[CR37] Southwick EE (1983). 1983 The honey bee cluster as a homeothermic superorganism. Comp Biochem Physiol Part A Physiol.

[CR38] Stabentheiner A, Kovac H, Mandl M, Käfer H (2021). Coping with the cold and fighting the heat: thermal homeostasis of a superorganism, the honeybee colony. J Comp Physiol A Neuroethol Sensory, Neural, Behav Physiol [Internet].

[CR39] Sudarsan R, Thompson C, Kevan PG, Eberl HJ (2012). Flow currents and ventilation in Langstroth beehives due to brood thermoregulation efforts of honeybees. J Theor Biol Internet.

[CR40] Thompson C (2013). A Cfd study investigating the influence of bottom board geometry on physical processes within a standard honeybee hive. J Chem Inf Model.

[CR41] Venkatesh V (2016) CFD with OpenSource software tutorial of convective heat transfer in a vertical slot. Proc CFD with OpenSource Softw [Internet]. Available from: http://www.tfd.chalmers.se/~hani/kurser/OS_CFD_2016/VarunVenkatesh/Varun_report.pdf

[CR42] Watmough J, Camazine S (1995). Self-organized thermoregulation of honeybee clusters. J Theor Biol.

[CR43] Widenius UM (2020) MariaDB version 10.2.36

